# Cancer Cells Evade Stress-Induced Apoptosis by Promoting HSP70-Dependent Clearance of Stress Granules

**DOI:** 10.3390/cancers14194671

**Published:** 2022-09-25

**Authors:** Aifang Wang, Xianmixinuer Abulaiti, Han Zhang, Hang Su, Guangzhi Liu, Shaorong Gao, Lingsong Li

**Affiliations:** 1 Shanghai Advanced Research Institute, Chinese Academy of Sciences, Shanghai 201210, China; 2 University of Chinese Academy of Sciences, Beijing 100049, China; 3 Clinical and Translational Research Center of Shanghai First Maternity & Infant Hospital, Frontier Science Center for Stem Cells, School of Life Sciences and Technology, Tongji University, Shanghai 200092, China; 4 Henan Key Laboratory of Stem Cell Differentiation and Modification, Henan Provincial People’s Hospital, Academy of Medical Science, Zhengzhou University, Zhengzhou 450053, China

**Keywords:** proteostasis, stress granule dynamics, chaperone protein HSP70, tumorigenesis, apoptosis

## Abstract

**Simple Summary:**

The formation of stress granules is a cellular mechanism to limit protein synthesis and avoid the production of unfolded proteins in stressed cells. In the present study, we found that prolonged stress caused persistent stress granules, leading to cell death in normal cells. However, cancer cells can evade stress-induced cell death by promoting HSP70-dependent clearance of stress granules. In other words, the dynamics of stress granules determine the cell status during prolonged stress. Our work provides insight into tumorigenesis in stressed cells. It also suggests a new approach to potentially treating cancers by modulating the dynamics of stress granules.

**Abstract:**

The formation of stress granules (SG) is regarded as a cellular mechanism to temporarily limit protein synthesis and prevent the unfolding of proteins in stressed cells. It has been noted that SG formation can promote the survival of stressed cells. Paradoxically, however, persistent SGs could cause cell death. The underlying molecular mechanism that affects the relationship between SG dynamics and cellular states is not fully understood. Here we found that SG dynamics in cancer cells differ significantly from those in normal cells. Specifically, prolonged stress caused the formation of persistent SGs and consequently resulted in apoptosis in the normal cells. By contrast, cancer cells resolved SGs and survived the prolonged stress. Regarding the mechanism, the knockdown of HSP70 or the inhibition of the HSP70s’ ATPase activity caused defective SG clearance, leading to apoptosis in otherwise healthy cancer cells. On the other hand, the knockout of G3BPs to block the formation of SGs allowed cancer cells to escape from the HSP70 inhibition-induced apoptosis. Given the observation that SG dynamics were barely affected by the inhibition of autophagy or proteasome, we propose that SG dynamics are regulated mainly by HSP70-mediated refolding of the unfolded proteins or their removal from SGs. As a result, cancer cells evade stress-induced apoptosis by promoting the HSP70-dependent SG clearance.

## 1. Introduction

The formation of stress granules (SGs) is a cellular mechanism to limit protein synthesis by the ribosome and avoid the production of unfolded proteins in stressed cells [1,2]. It has been proposed that the SG formation provides pro-survival signals in cells under stress [3,4,5]. Paradoxically, however, persistent SGs are associated with cell death [6,7]. In this regard, SGs serve as a part of the proteostasis network. In other words, the balance between the assembly and the disassembly of SGs can regulate protein homeostasis and cellular status in cells under stress [3,8].

The assembly of SGs is mediated by liquid–liquid phase separation (LLPS), which is mainly regulated by G3BP1. The latter is a GAP SH3-binding protein [9]. Upon stress loads, G3BP1 triggers the formation of SGs, which are membrane-less organelles containing multiple RNA-binding proteins, including G3BP1 [9,10]. In addition, SGs also contain defective ribosomal products (DRiPs), which are unfolded or misfolded at the ribosome during stress [11]. On the other hand, the disassembly of SGs depends on the removal of G3BP1 and DRiPs from SGs [11,12,13]. In this case, G3BP1 is ubiquitinated and then removed from SGs by the interaction among the endoplasmic reticulum-associated protein FAF2 and the ATPase valosin-containing protein (VCP) [11,13]. For temporary stress, DRiPs are removed from SGs by the heat shock protein 70 (HSP70), which is a chaperone protein essential for the refolding of proteins by hydrolyzing adenosine triphosphate (ATP) [2,11,14].

It has been proposed that if DRiPs cannot be removed from SGs, the proteasome system will be then activated to degrade misfolded proteins, or, alternatively, the autophagy system would be activated for the clearance of SGs [11,15]. However, these proposals have not been fully verified. For example, it has been reported that the inhibition of autophagy barely affected the clearance of SGs in cells during prolonged stress [2]. It is worth noting that most of the studies on SG dynamics have been performed in cancer cells, while basal autophagy levels are low in normal cells but much high in cancer cells [16]. From this perspective, the relationship between SG dynamics and cellular states in normal versus cancer cells was thoroughly investigated. The hypothesis that cancer cells evade stress-induced apoptosis by promoting HSP70-dependent clearance of SGs was also further tested.

## 2. Materials and Methods

### 2.1. Cell Lines and Cell Cultures

HeLa, SH-SY5Y, 293T, and U2OS cells were all purchased from the National Collection of Authenticated Cell Cultures (Shanghai, China). They were cultured in DMEM-high glucose (Gibco, Carlsbad, CA, USA), which was supplemented with 10% (*v*/*v*) fetal bovine serum (FBS, Gibco, Carlsbad, CA, USA) and 1% penicillin/streptomycin at 37 °C in 5% CO_2_. In addition, human embryonic stem cells (ESCs) H9 were obtained from Dr. Hongkui Deng, Peking University Stem Cell Research Center (Beijing, China) [17]. Coat plates with 1% matrigel (354277, Corning, New York, NY, USA) were heated at 37 °C for 1 h, cultured in mTeSR™1 medium (85850, STEMCELL, Vancouver, Canada), and supplemented with 1% penicillin/streptomycin and Y27632 (10 μM).

### 2.2. Primary Neural Progenitor Cells Cultures

The C57 mouse primary Neural Progenitor Cells (NPCs) at E15.5 were obtained according to the previously reported methods, with some modifications [18]. Coat coverslips with 1% matrigel (354277, Corning, New York, USA) were heated at 37 °C for 1 h. Mice brains were removed immediately after anesthesia and decapitation under sterile conditions. Next, the meninges were removed, the cortex was isolated, rinsed with HBSS, finely minced into small pieces, and then incubated with 0.25% trypsin solution for 5 min at 37 °C. After the trypsinization and washing processes, the cell suspension was filtered through a 70 μm cell strainer, while cell debris and big tissues were discarded. The cell suspension was centrifuged at 1500 rpm for 5 min, the supernatant was discarded, and the cell pellet was suspended in a culturing medium (DF12 medium supplemented with 1% N2 supplement (17502048, Gibco, Carlsbad, CA, USA), L-glutamine (0.5 mM), 1% penicillin/streptomycin, 20 ng/mL bFGF, 20 ng/mL EGF), and finally the cells were returned to the incubator (5% CO_2_, 37 °C). The NPCs were passaged with Accutase, and the fresh culture medium was replaced every 2 days.

### 2.3. Primary Cortical Neuron Cultures

The C57 mouse primary cortical neurons at E15.5 were obtained according to the previously reported methods, with some modifications [19]. More specifically, coverslips were coated with 0.15 mg/mL poly-D-lysine overnight. Afterward, mice brains were removed immediately after anesthesia and decapitation under sterile conditions. Next, the meninges were removed, the cortex was isolated, rinsed with HBSS, finely minced into small pieces, and then incubated with 0.25% trypsin solution for 15 min at 37 °C. After the trypsinization and washing processes, the cell suspension was filtered through a 70 μm cell strainer, while cell debris and big tissues were discarded. The cell suspension was centrifuged at 1500 rpm for 5 min, the supernatant was discarded, and the cell pellet was suspended in a plating medium (MEM was supplemented with 0.6% glucose, 2 mM glutamine, and 10% FBS). The cells (5% CO_2_, 37 °C) were incubated for 3 h, the plating medium was removed and replaced with culturing medium (MEM was supplemented with 0.6% glucose, 2 mM glutamine, 1 mM pyruvate, 0.1% BSA, and 1% N2 supplement). Finally, the cells were returned to the incubator (5% CO_2_, 37 °C).

### 2.4. Immunofluorescence Staining

Immunofluorescence staining was performed according to the previously reported methods, with some modifications [20]. The cells were fixed in 4% paraformaldehyde (PFA, HT501128, Sigma, Darmstadt, Germany) for 30 min at 4 °C, and then the cells were blocked with a blocking solution (DPBS containing 0.3% Triton X-100 and 5% Bovine Serum Albumin) at room temperature. In addition, the coverslips were incubated with primary antibody overnight at 4 °C in the following dilutions: anti-G3bp1 (1:1000, ab181150, Abcam, Cambridge, UK), anti-G3bp1 (1:1000, ab56574, Abcam, Cambridge, UK), anti-G3bp2 (1:1000, NBP1-82977, Novus biologicals, Abingdon, UK), anti-CPARP (1:500, 9548, CST, Danvers, MA, USA), anti-SOX2 (1:1000, ab97959, Abcam, Cambridge, UK), anti-Ubiquitin (1:200, sc-8017, Santa Cruz, Dallas, TX, USA), anti-DCP1A (1:500, H00055802-M06, Abnova, Taipei, China), anti-HSP70 (1:1000, ab45133, Abcam, Cambridge, UK), anti-VCP (1:1000, ab109240, Abcam, Cambridge, UK), and anti-eIF3η (1:200, sc-137214, Santa Cruz, Dallas, TX, USA). After being washed 3 times in PBS, the coverslips were then incubated with Alexa Fluor 488, and 594 (Invitrogen, Carlsbad, CA, USA) secondary antibodies (at 1:500 dilutions) for 60 min at room temperature. Next, they were counterstained with Hoechst 33342 (Invitrogen, Carlsbad, CA, USA), washed, and mounted on slides by using a CC mount (Sigma, Darmstadt, Germany). The fluorescent signals were detected by using a Zeiss 710 confocal microscope or OlyVIA VS120 slide scanner and the images were processed with the ImageJ software version 1.53 (Bethesda, MD, USA).

### 2.5. Western Blotting (WB)

WB was performed according to the previously reported methods, with some modifications [21]. Cells were harvested in RIPA lysis buffer (P0013B, Beyotime, Shanghai, China) that was supplemented with complete protease inhibitors (Sigma, Darmstadt, Germany). Cell lysates were separated on 10% SDS-PAGE and blotted onto the NC membrane. The membrane was then blocked in 5% nonfat milk and incubated with the primary antibodies overnight at 4 °C in the following dilutions: anti-G3bp1 (1:3000, ab181150, Abcam, Cambridge, UK), anti-G3bp1 (1:3000, ab56574, Abcam, Cambridge, UK), anti-G3bp2 (1:3000, NBP1-82977, Novus biologicals, Abingdon, UK), anti-Ubiquitin (1:1000, sc-8017, Santa Cruz, Dallas, TX, USA), anti-HSP70 (1:10000, ab45133, Abcam, Cambridge, UK), anti-VCP (1:3000, ab109240, Abcam, Cambridge, UK), anti-LC3 (1:1000, A19665, ABClonal, Wuhan, China), and anti-GAPDH (1:10000, KC-5G5, Kangchen Biotech, Shanghai, China). After the membranes were washed with 0.1% PBST, HRP-conjugated anti-mouse (7076S, CST, Danvers, MA, USA) or anti-rabbit (7074S, CST, Danvers, MA, USA) antibody was used as the secondary antibody for 1 h at room temperature. The signals were detected by using the Western ECL Substrate (170-5060, Bio-Rad, Irvine, CA, USA). The images were visualized by using the Bio-Rad ChemiDoc XRS+ system. 

### 2.6. Fluorescence Recovery after Photobleaching (FRAP)

FRAP experiments were performed by using a Nikon CSU-W1 microscope equipped with 60X oil immersion objectives as has been previously described, with some modifications [13,22]. The cells were seeded into a 35 mm confocal dish with a 20 mm bottom well (D35-20-1.5-N, Cellvis, Mountain View, CA, USA) 48 h prior to imaging. The cells were transfected with pCMV7.1-G3BP1-GFP 24 h prior to imaging. For FRAP in the cells, time-lapse images were collected every 1 s for 3 min by using 488-nm imaging lasers set at 200 ms exposure. The applied photobleaching settings were the following: 100 ms, 70% of 405 channels. The acquired images were analyzed by using the NIS Elements software. In Nikon Elements, ROIs were generated in the photobleached region, as well as a nonphotobleached cell, and the background for each time-lapse, whereas the mean intensity of each was extracted. For the photobleached regions, a 3-μm-diameter circle was used. The data were repeated in triplicate for each condition, with each replicate processing at least n = 15 cells. During the imaging process, the cells were maintained at 42 °C and supplied with 5% CO_2_ by using a Bold Line Cage Incubator (Okolabs) and an objective heater (Bioptechs).

### 2.7. Heat Shock and Drug Treatments

For the heat shock experiment, the cells were transferred to a 42 °C humidified incubator with 5% CO_2_. The samples were collected according to the different heat shock time points marked in the figure. As far as drug treatments are concerned, 2-DG was dissolved in the DMEM medium, and other chemicals were dissolved in DMSO, which was added to the cells as follows: 2-DG (100 mM, S4701, Selleck, Houston, TX, USA), VER155008 (40 μM, 70 μM, 100 μM, S7751, Selleck, Houston, TX, USA), Bafilomycin A1 (10 nM, 100 nM, 1 μM, HY-100558, MCE, New Jersey, USA), and MG132 (10 μM) were dissolved respectively in the culture media and prewarmed before the treatment. When cells were treated with VER155008 or 2-DG, added after 1 h of heat shock at 42 °C, the samples were collected at the indicated time points. When the cells were treated with Bafilomycin A1, they were pretreated for 18 h at 37 °C before the heat shock experiments were performed [13], and the samples were collected at the indicated time points.

### 2.8. siRNA Transfection

For the knockdown HSP70 experiment, the siRNA oligos were purchased from Genepharma (Suzhou, China), and then transfected into HeLa cells as has been previously described [23,24]. Twenty-four h before the transfection, HeLa cells were trypsinized to obtain 40–50% confluency on the day of transfection. For a 35 mm dish, 5 μL of Lipofectamine 2000 (Invitrogen, Carlsbad, CA, USA) was diluted in a 200 μL Opti-MEM medium. Next, they were mixed and incubated for 5 min. Subsequently, 100 pmol siRNA was diluted in 200 μL Opti-MEM medium, and the diluted siRNA was added to the diluted Lipofectamine 2000 reagent (1:1 ratio), where it was mixed and incubated for 20 min. Then, the oligomer-Lipofectamine 2000 complex was added to the cells. The siRNA was incubated for 36 h before the experiments. The siRNA oligos used in this work are listed below:

si-Ctrl: 5′-UUCUCCGAACGUGUCACGUTT-3′

si-HSP70-1#: 5′-CCAAGCAGACGCAGAUCUUTT-3′

si-HSP70-2#: 5′-CGGUUUCUACAUGCAGAGATT-3′

### 2.9. RNA Isolation and qRT-PCR

RNA isolation and real-time quantitative RT-PCR were performed according to the previously reported methods, with some modifications [25] The total RNA was extracted from cells by using the MiniBEST Universal RNA Extraction Kit (Takara, Kyoto, Japan). In addition, RT-PCR was performed by using the PrimeScript RT Master Mix (Takara, Kyoto, Japan). The expression of the genes was detected by qRT-PCR (Applied Biosystems, Carlsbad, CA, USA) by using SYBR Premix Ex Taq II (Takara, Kyoto, Japan). The expression of the genes was also normalized to that of the internal control *Gapdh*. The data were analyzed by ANOVA with multiple comparisons of the means in GraphPad Prism 8.0 (San Diego, CA, USA). The acquired results of qRT-PCR are provided as the mean ± SEM from three independent biological replicates. Primer sequences are listed in Table S1.

### 2.10. ATP Measurement

The intracellular ATP levels were determined by using an ATP Bioluminescence Assay kit (S0026, Beyotime, Shanghai, China) according to the manufacturer’s protocol. The protein concentrations were estimated by using the BCA protein assay kit (23225, Thermo Scientific, Waltham, MA, USA). The ATP content was expressed as μmol/g protein, and the data are presented as the average of the three independent experiments. An amount of 100 mM 2-DG (S4701, Selleck, Houston, TX, USA) was also used to inhibit the glycolysis pathway [13].

### 2.11. CRISPR-Cas9-Mediated Knockout Cells

The G3BP1 and G3BP2 double-KO HeLa cells were designed according to the previously reported method, with a few modifications [26]. The gRNA for G3BP1 or G3BP2 was designed through http://crispor.tefor.net/ (accessed on 20 May 2020) and the synthesized DNA oligos were ligated into BbsI (NEB)-digested px459 vectors (62988, Addgene, Watertown, MA, USA) [27]. The targeting vectors were verified by Sanger sequencing. The WT HeLa cells were transiently transfected with two gRNA vectors targeted for G3BP1 by using Lipofectamine 2000 overnight, allowed to recover for about 2 days, which was followed by the addition of 2 μg/mL puromycin for 5 days. The G3BP1 knockout clones were first enriched to obtain the pure cell line by the dilution of cells in 24-well plates. The successful knockout was verified by antibody staining and Western blot. Moreover, to create the double-KO ΔΔG3BP1/2 cell line, the ΔG3BP1 cells were then transfected with two gRNA vectors targeted for G3BP2, whereas the subsequent procedure to obtain G3BP2 knockout cells was the same as that for G3BP1. The ΔΔG3BP1/2 cell line was also verified by antibody staining and Western blot analysis. The DNA oligos for gRNA are listed in Table S1.

### 2.12. Statistical Analyses

All statistical analyses were performed by using the statistical software GraphPad Prism (version 8.0, San Diego, CA, USA) with ANOVA or Student’s *t*-test. The data are presented as mean ± SEM.

## 3. Results

### 3.1. Apoptosis Occurs in ESCs but Not in HeLa Cells during Prolonged Stress

**Figure 1:**
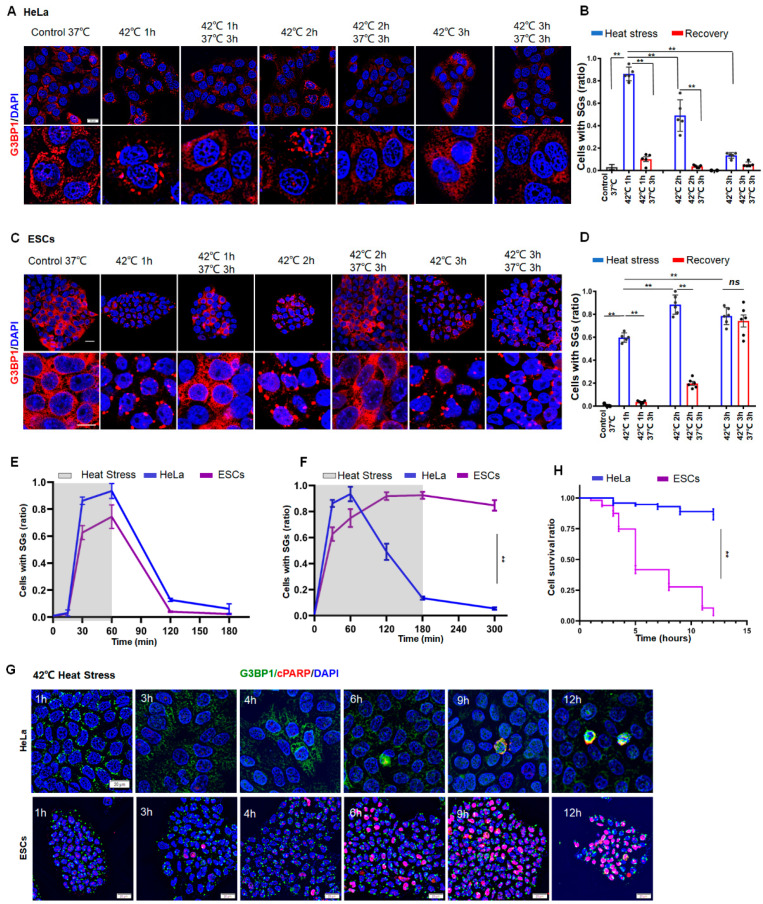
SG Dynamics and cell death in HeLa or ESCs under prolonged stress. (**A**,**B**) SGs in HeLa cells under 42 °C stress for 1, 2, or 3 h, respectively. Scale bar, 20 μm. Mean ± SEM. ** *p* < 0.01 (ANOVA test). (**C**,**D**) SGs in ESCs under 42 °C stress for 1, 2, or 3 h, respectively. Scale bar, 20 μm. Mean ± SEM. ** *p* < 0.01 (ANOVA test). (**E**) Statistical analysis of G3BP1-SGs in HeLa cells or ESCs after 42 °C stress for 1 h then recovery for 2 h at 37 °C. Mean ± SEM. (**F**) Statistical analysis of G3BP1-SGs in HeLa cells or ESCs after 42 °C stress for 3 h, followed by recovery for 2 h at 37 °C. Mean ± SEM. ** *p* < 0.01 (ANOVA test). (**G**,**H**) cPARP staining and cell survival ratio in HeLa or ESCs under prolonged stress. Scale bar, 20 μm. Mean ± SEM. ** *p* < 0.01 (ANOVA test).

To investigate the difference in SG dynamics between normal and cancer cells, we subjected human ESCs or human cervical carcinoma (HeLa) cells to heat stress at 42 °C for 1, 2, or 3 h, respectively. The granule numbers were then measured in the cells by staining of G3BP1, which is an essential mediator for the formation of SGs. As a result, SG numbers reached a peak after 1 h in HeLa cells (Figure 1A,B), whereas this was 2 h in ESCs (Figure 1C,D). If the stress was removed before 2 h, SGs eventually disappeared in both HeLa (Figure 1B, left two panels) and ESCs (Figure 1D, left two panels). If the cells were subjected to continuous stress for 3 h, however, the SGs in ESCs plateaued during persistent stress (Figure 1D, right panel). By contrast, the existence of SGs in HeLa cells could no longer be observed (Figure 1B, right panel). Similarly, the disappearance of SGs during prolonged stress was also observed in other cancer cells, such as 293T (SV40 T antigen-transformed human embryonic kidney) and U2OS (human osteosarcoma epithelial) cells (Figure S1A–C). Despite no significant differences between HeLa and ESCs during 1 h stress (Figure 1E), the SG dynamics in ESCs significantly differed from those in the cancer cells under prolonged stress (Figure 1F). Moreover, half of the ESCs died around 6 h (Figure 1G,H, lower panel and purple; Figure S1D), whereas HeLa cells managed to survive the prolonged stress (Figure 1G,H, upper panel and blue), as indicated by staining of TUNEL and cleaved poly (ADP-ribose) polymerase (c-PARP). A curve of the SG dynamics (Figure 1F) was inversely correlated with a curve of the cell death (Figure 1H) in both HeLa and ESCs. These data suggested that cancer cells can resolve SGs and escape from stress-induced cell death, whereas normal cells failed to clear SGs and thus cannot survive under prolonged stress.

### 3.2. HeLa Cells Can Resolve SGs While ESCs Fail to Do So Due to Low Levels of HSP70

**Figure 2:**
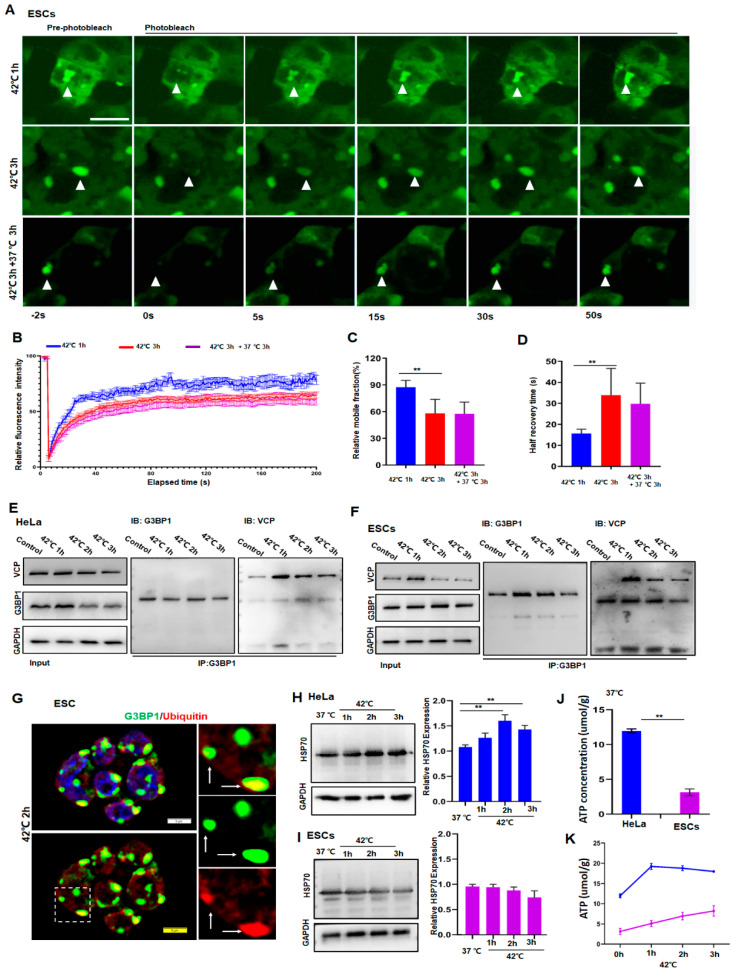
HeLa cells resolved SGs but ESCs failed to do so due to low levels of HSP70 and ATP. (**A**,**B**) FRAP analysis of G3BP1-SGs in ESCs after 42 °C stress for 1 or 3 h and then recovery for 3 h at 37 °C. n = 15 cells for each group. Mean ± SEM. Scale bar, 10 μm. (**C**,**D**) Quantification of mobile fraction and half recovery time in ESCs after 42 °C stress for 1 or 3 h then recovery for 3 h at 37 °C. n = 15 cells for each group. Mean ± SEM. ** *p* < 0.01 (ANOVA test). (**E**) Immunoblotting of VCP after immunoprecipitation of G3BP1 from HeLa cells after 42 °C stress for the indicated time. (**F**) Immunoblotting of VCP after immunoprecipitation of G3BP1 from ESCs after 42 °C stress for the indicated time. (**G**) G3BP1 and ubiquitin staining in ESCs under 42 °C stress for 2 h. Scale bar, 5 μm. (**H**,**I**) HSP70 in HeLa or ESCs under 42 °C stress for the indicated time. Mean ± SEM. ** *p* < 0.01 (ANOVA test). Original blots see Supplementary File S1. (**J**,**K**) ATP concentrations in HeLa or ESCs after 42 °C stress for the indicated time. Mean ± SEM. ** *p* < 0.01 (ANOVA test).

To understand the reason for ESCs containing persistent SGs, we investigated whether proteins in SGs were subjected to a liquid-to-solid transition under prolonged stress. For that reason, we introduced G3BP1-tagged with green fluorescent protein (GFP) in ESCs and then examined the G3BP1 mobility by fluorescence recovery after photobleaching. As shown in Figure 2, despite a reduction in its mobility in ESCs after 3 h of stress (Figure 2A–D, red versus blue), G3BP1 did not yet undergo a liquid-to-solid transition under these conditions. Hence, it can be argued that persistent SGs may result from a failure of the removal of G3BP1 or DRiPs from SGs.

The clearance of G3BP1 and DRiPs requires the existence of ubiquitin-proteasome molecules such as VCP and chaperone proteins such as HSP70 [2,28,29,30]. Therefore, the expression of VCP, ubiquitin, G3BP1, and HSP70 in HeLa and ESCs was examined. As can be ascertained from Figure 2, the interaction between VCP and G3BP1 had no obvious differences compared to the respective interaction between HeLa and ESCs as analyzed by co-immunoprecipitation of the related proteins in HeLa (Figure 2E) and ESCs (Figure 2F). Moreover, the co-localization of G3BP1 and ubiquitin in granules (Figure 2G) suggested that, as was reported in HeLa cells [13], the ubiquitin–VCP system in ESCs appeared to interact normally. Additionally, to functionally verify these observations, autophagy was blocked by bafilomycin A1 (BafA1), an inhibitor of lysosomal V-ATPase [31], while the proteasome pathway was inhibited by MG132 [32]. We then compared SG dynamics and the cellular status in ESCs and Hela cells. As speculated, SGs disappeared in HeLa cells but plateaued in ESCs after the application of 3 h of stress, despite the addition of BafA1 (Figure S2A,B) or MG132 (Figure S2C) to the cultured cells. Accordingly, apoptosis was only observed in ESCs but not in HeLa cells under these stress conditions (Figure S2D). These data clearly indicated that the inhibition of either autophagy or the proteasome pathway did not influence the dynamics of SGs. In other words, the clearance of SGs appeared to be little affected by autophagy or the proteasome pathway.

After the relationship between SG dynamics and these pathways governing protein homeostasis was calculated, the role of HSP70 in the regulation of SG dynamics in normal and cancer cells was investigated. First, the expression of *HSP70* was significantly higher in HeLa cells than in ESCs (Figure S2E). Moreover, in a response to heat stress, the production of HSP70 was significantly upregulated in the cancer cells in a time-dependent manner (Figure 2H and Figure S2F,G) but was barely changed in the normal cells (Figure 2I and Figure S2F,G). As a partner that is essential for the functioning of HSP70s, the cellular ATP concentrations were also significantly higher in HeLa cells (blue) than in ESCs measured at any time during the stress (Figure 2J,K). These data suggest that the differences in the dynamics of SGs between HeLa and ESCs may be due to the upregulation of HSP70, which is required for the refolding of unfolded proteins at the cost of ATP.

### 3.3. Neuroblastoma (SY5Y) Cells Can Radically Resolve SGs While Normal Neural Cells Cannot

**Figure 3:**
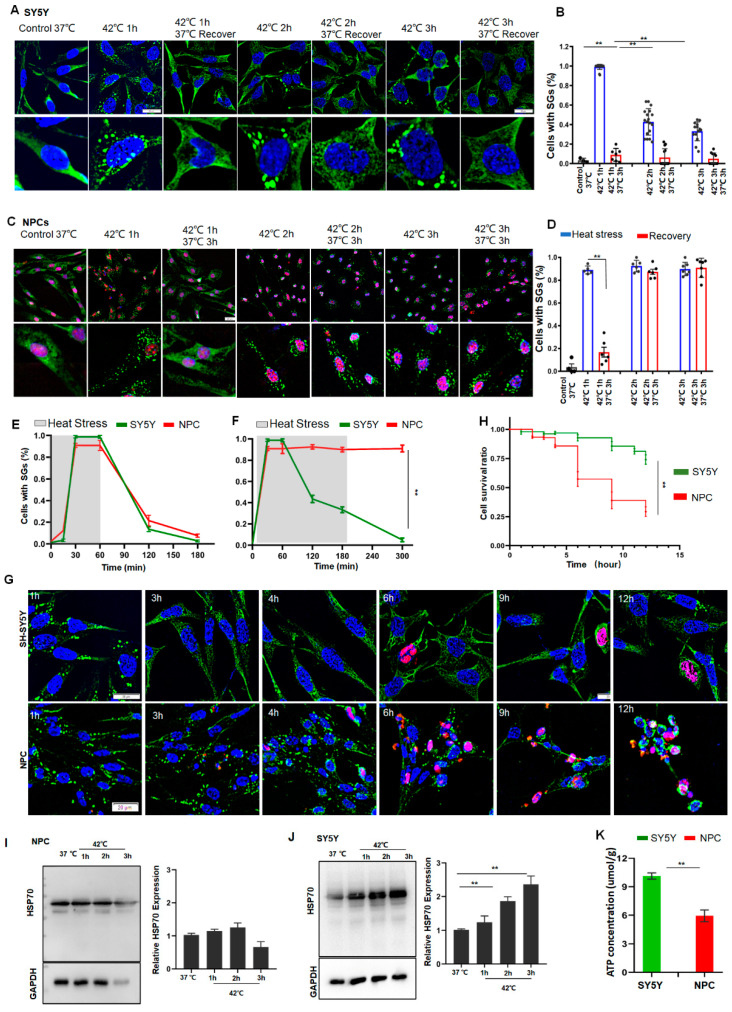
SY5Y cells resolved SGs but NPCs failed to do so due to low levels of HSP70 and ATP. (**A**,**B**) SGs in SY5Y cells under 42 °C stress for 1, 2, or 3 h, respectively. Scale bar, 20 μm. Mean ± SEM. ** *p* < 0.01 (ANOVA test). (**C**,**D**) SGs in NPCs (G3BP1 and SOX2 staining) under 42 °C stress for 1, 2, or 3 h, respectively. Scale bar, 20 μm. Mean ± SEM. ** *p* < 0.01 (ANOVA test). (**E**) SGs in SY5Y cells or NPCs after 42 °C stress for 1 h followed by recovery for 2 h at 37 °C. Mean ± SEM. (**F**) SGs in SY5Y cells or NPCs after 42 °C stress for 3 h followed by recovery for 2 h at 37 °C. Mean ± SEM. ** *p* < 0.01 (ANOVA test). (**G**,**H**) cPARP staining and cell survival ratio in SY5Y cells or NPCs under prolonged stress. Scale bar, 20 μm. Mean ± SEM. ** *p* < 0.01 (ANOVA test). (**I**,**J**) HSP70 in NPCs or SY5Y cells after 42 °C stress for the indicated time. Mean ± SEM. ** *p* < 0.01 (ANOVA test). Original blots see Supplementary File S1. (**K**) ATP concentrations in SY5Y cells or NPCs. Mean ± SEM. ** *p* < 0.01 (Students *t*-test).

To further determine whether these differences in SG dynamics between ESCs and HeLa cells under prolonged stress are a common phenomenon between normal and cancer cells, we examined SG dynamics, cell survival, HSP70, and ATP levels in neuroblastoma SY5Y cells, NPCs, and primary cultured neurons. As can be seen from Figure 3, during 1 h of short stress, the number of SGs peaked at 1 h after stress and gradually disappeared over time in either SY5Y or NPCs (Figure 3A–E). If the stress persisted over 3 h, scarcely any SG was observed in SY5Y cells (Figure 3B,F) and the cells managed to survive in these stress conditions. By contrast, the SG numbers in NPCs plateaued (Figure 3D,F), and half of the cells died after 6 h stress (Figure 3G,H). As expected, the SG dynamics and cellular status in the primary cultured neurons behaved similarly to NPCs (Figure S3A–C). In line with these observations in HeLa cells and ESCs, the HSP70 levels were negligibly changed in the normal NPCs (Figure 3I), but were significantly increased in the cancer SY5Y cells in a time-dependent manner (Figure 3J). Accordingly, the ATP levels in SY5Y cells were significantly higher than those in NPCs (Figure 3K). Based on these data, it can be concluded that the upregulation of HSP70 may represent a common characteristic of most cancer cells under stress, whereas this upregulation may effectively protect cancer cells from apoptosis by the clearance of SGs during prolonged stress.

**Figure 4:**
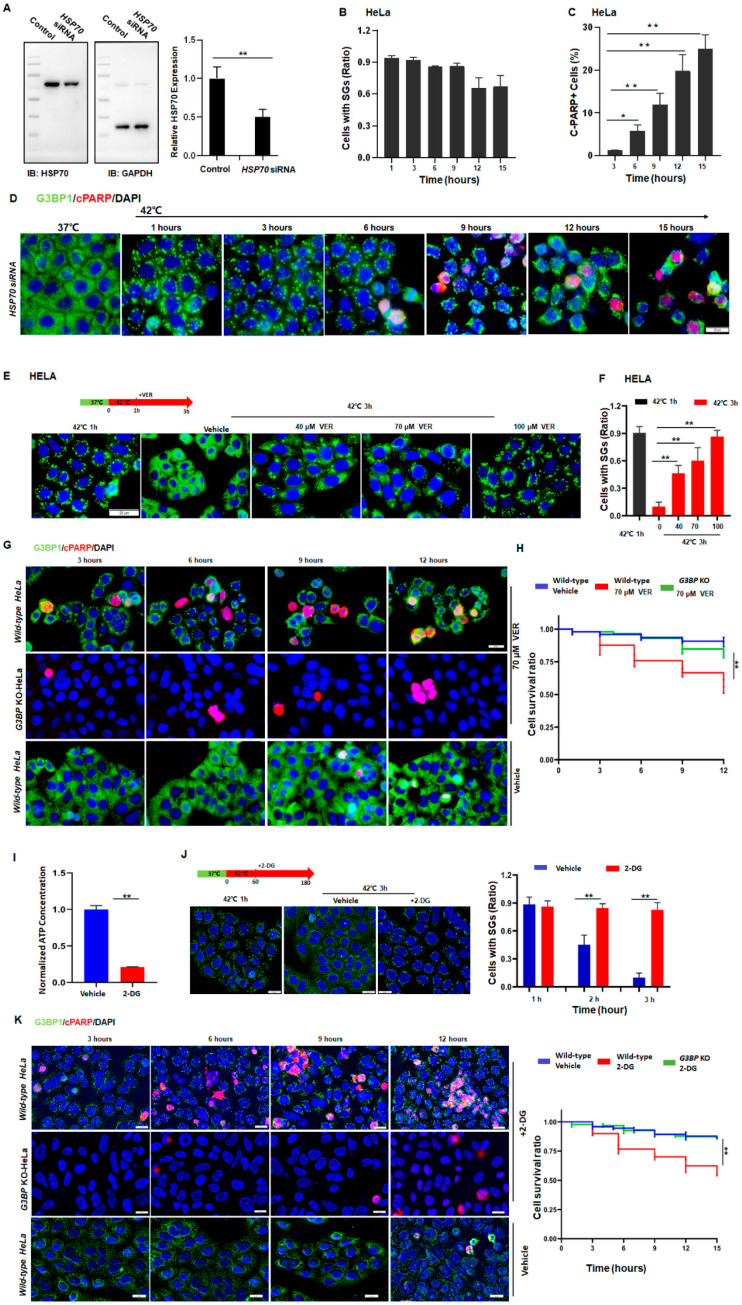
Cancer cells sneak under stress past the protein quality control system by promoting HSP70-dependent SG clearance. (**A**) HSP70 in HELA cells treated with control or *HSP70* siRNA. Mean ± SEM. ** *p* < 0.01 (Student’s *t*-test). (**B**) SGs ratio in HeLa cells treated with *HSP70* siRNA exposed to 42 °C stress for the indicated time. Mean ± SEM. No significance (ANOVA test). (**C**,**D**) cPARP staining in HeLa cells treated with *HSP70* siRNA exposed to 42 °C stress for the indicated time. Scale bar, 20 μm. Mean ± SEM. * *p* < 0.05, ** *p* < 0.01 (ANOVA test). (**E**,**F**) SGs in HeLa cells treated with or without VER exposed to 42 °C stress for 3 h. Scale bar, 20 μm. Mean ± SEM. ** *p* < 0.01 (ANOVA test). (**G**,**H**) cPARP staining in HeLa cells treated with or without VER exposed to 42 °C stress for the indicated time. Scale bar, 20 μm. Mean ± SEM. ** *p* < 0.01 (ANOVA test). (**I**) ATP levels in HeLa cells treated with or without 100 mM 2-deoxy-D-glucose (2-DG) for 2 h from three biological replicates. Mean ± SEM. ** *p*< 0.01 (Student’s *t*-test). (**J**) SGs in HeLa cells after 42 °C stress for 3 h with or without 100 mM 2-DG for 2 h. Scale bar, 20 μm. Mean ± SEM. ** *p* < 0.01 (Student’s *t*-test). (**K**) cPARP staining in HeLa cells during prolonged stress conditions with or without 100 mM 2-DG treatment. Scale bar, 20 μm. Mean ± SEM. ** *p* < 0.01 (ANOVA test).

To further verify the role of HSP70 in the regulation of SG dynamics, HSP70 was knocked down in the cells (Figure 4A and Figure S4A) and the dynamics of SGs were tracked. As has been demonstrated, after HSP70-knockdown, these HeLa (Figure 4B,D) and SY5Y (Figure S4B,D) cancer cells retained high levels of SG numbers. By contrast, regular cancer cells resolved SGs under the same conditions (Figure 1A,B and Figure S1A–C). Interestingly, these HSP70-knockdown cancer cells can no longer survive, indicated by the significant increase in c-PARP+ cells in both HeLa (Figure 4C,D) and SY5Y (Figure S4C,D) cells. Evidently, HSP70 is essential for the clearance of SGs, suggesting that persistent SGs may cause cell death in stressed cells.

### 3.4. Cancer Cells under Stress Evade Stress-Induced Apoptosis by Promoting HSP70-Dependent SG Clearance

To further investigate the relationship among HSP70, SG dynamics, and apoptosis, G3BP1 and G3BP2 from HeLa cells were genetically knocked out to generate cells (Figure S5A–C) unable to form SGs under most stress conditions [9]. By using these cells, we first determined the chemical VER concentration such that it could efficiently inhibit the HSP70 activity but barely have an impact on cell survival (Figure S5D,E). The hypothesis that SG dynamics can directly determine the cellular status in the cells under stress was then tested.

As expected, the inhibition of HSP70 by adding VER caused defective clearance of SGs in a dose-dependent manner (Figure 4E,F), leading to apoptosis in about 50% of the wild-type HeLa cells after 12 h of heat stress (Figure 4G,H). In addition, a reduction in ATP levels also blocked the clearance of SGs (Figure 4I,J) and led to cell death in otherwise surviving cancer cells (Figure 4K). Similar results were also observed in SY5Y cells (Figure S4E–I). On the other hand, such effects of HSP70-inhibition (Figure 4G,H, upper panels, green) or downregulation of ATP (Figure 4K, upper panels, green) were no longer observed in the G3BP-knockout HeLa cells (Figure 4K). Based on these data, it was confirmed that persistent SGs can also cause apoptosis in cancer cells under prolonged stress. Moreover, cancer cells can upregulate the expression of HSP70 to resolve SGs and evade stress-induced apoptosis in the face of stress.

## 4. Discussion

The formation of SGs serves as a part of the proteostasis network to limit protein synthesis and avoid producing unfolded proteins by ribosomes in a response to stress. As such, the dynamics of SGs determine the degree of functional recovery after stress relief or cell death during persistent stress.

It has been documented that the clearance of SGs involves multiple molecular pathways including HSP70, the ubiquitin–VCP proteasome pathway, and autophagy [2,11,28,33]. However, a discrepancy has been reported that either inhibition of autophagy or proteasome pathway had a negligible impact on the clearance of SGs [2]. In the present study, we also found that, despite autophagy being indeed inhibited as indicated by the increased LC3-II expression [34] (Figure S2B), BafA1 did not affect the SG dynamics (Figure S2A). Likewise, the inhibition of the proteasome pathway by MG132 did not affect the clearance of SGs (Figure S2C). By considering that the inhibition of HSP70 could block half of SG clearance in either HeLa (Figure 4) or SY5Y cells (Figure S4), it can be concluded that HSP70 plays a major role in the clearance of SGs and the regulation of SG dynamics.

Our conclusion fully agrees with the report by Ganassi et al. [2]. In that report, the authors proposed that DRiPs in SGs are initially recognized by heat shock protein B8 (HSPB8), which recruits BAG3 (Bcl-2-associated athanogene 3) and HSP70 to disassemble SGs by clearing DRiPs. Despite the agreement on the HSP70 function in the regulation of SG dynamics, nevertheless, how HSP70 clears DRiPs remains to be ambiguous. We propose that HSP70-mediated refolding or removing of DRiPs from SGs may account for the clearance of DRiPs. This proposal is partially supported by the observation that, after the disassembly of SGs, HSP70 was colocalized with DRiPs in the aggresome [2]. Regardless of the mechanism underlying this HSP70 function, the accumulated studies indicate that aberrant SG dynamics evolve in the pathology of human diseases [35]. In the present study, we uncovered that, unlike in normal cells where prolonged stress caused persistent SGs and consequently cell death, cancer cells could escape from stress-induced apoptosis by promoting HSP70-mediated clearance of SGs.

The difference between SG dynamics in normal cells and cancer cells provides not only a mechanism for HSP70-mediated regulation of SG dynamics, but also a plausible mechanism for a long-standing medical question: why there exists an inverse association between cancers and neurological degenerative disorders such as Alzheimer’s disease (AD) [36]. For instance, in AD patients’ neurons, chronic stress in aging could cause persistent SGs that lead to neuronal death, the main pathological feature of Alzheimer’s [37]. On the other hand, oncogenic factors may induce an aberrant expression of HSP70 in the stressed neurons. Such HSP70-aberrant neurons could overcome stress-induced apoptosis, leading eventually to tumorigenesis. This hypothesis for stress-induced tumorigenesis merits further investigation.

There are always more questions than answers. For example, it has been reported that SGs induced by distinctive stresses can be removed by different molecular pathways [11,12,13]. Does HSP70 also play an essential role in the clearance of SGs induced by stress other than heat? We are not sure at this moment, but it appeared that the clearance of MG132-induced SGs also exhibits a dependence on HSP70 (Figure S5F). Another crucial question is regarding the molecular mechanism underlying the HSP70-mediated clearance of SGs. Given the fact the inhibition of autophagy or proteasome pathways had little effect on SG clearance, we propose that refolding or removing of DRiPs from SGs by HSP70 may be the main reason for the clearance of SGs. Obviously, direct evidence for this proposal is needed in future studies.

## 5. Conclusions

Our study has demonstrated that SG dynamics determine the degree of functional recovery after stress relief or cell death during stress. Prolonged stress leads to persistent SGs and causes apoptosis in normal cells, but cancer cells can evade stress-induced apoptosis by promoting HSP70-dependent SG clearance. From this perspective, this work may provide a new approach to a potential treatment for neurodegenerative diseases by upregulating HSP70 expression in aging neurons. On the other hand, targeting HSP70 specifically in malignant cells might be helpful for treating cancers.

## Data Availability

The data that support the findings of this study are available from the corresponding author upon reasonable request.

## Supplementary Materials

The following supporting information can be downloaded at: https://www.mdpi.com/article/10.3390/cancers14194671/s1, Figure S1: Dynamics of SGs in 293T and U2OS cells under prolonged stress, Figure S2: The majority of SGs disassemble in HeLa cells despite inhibition of autophagy or proteasome under prolonged stress, Figure S3: Persistent SGs led to cell apoptosis in primary cultured neurons under prolonged stress, Figure S4: HSP70 inhibition leads to Persistent SGs and cell apoptosis in neuroblastoma under prolonged stress, Figure S5: Establishment of G3BPs knock out HeLa cell lines, File S1: Original Western Blot Data, Table S1: Resources table.
