# Supplementary material for: Cancer Cells Evade Stress-Induced Apoptosis by Promoting HSP70-Dependent Clearance of Stress Granules
Source: Cancers (Basel). 2022 Sep 25;14(19):4671. doi: 10.3390/cancers14194671 (PMC9562925; doi:10.3390/cancers14194671)
Supplement: Supplementary file 1 [file cancers-14-04671-s001.zip › cancers-1926987-supplementary.pdf]

Supplementary Information for

Cancer cells evade stress-induced apoptosis by promoting HSP70-dependent clearance of stress granules

Aifang Wang[1,2†], Xianmixinuer Abulaiti[1,3†], Han Zhang[1], Hang Su[4], Guangzhi Liu[4], Shaorong Gao[3*], Lingsong Li[1,2,4*]

*Correspondence: Lingsong Li (lils@sari.ac.cn) or Shaorong Gao (gaoshaorong@tongji.edu.cn)

This file includes:

Supplementary Figure

Original Western Blot Data

Supplementary Tables

Supplementary Figure

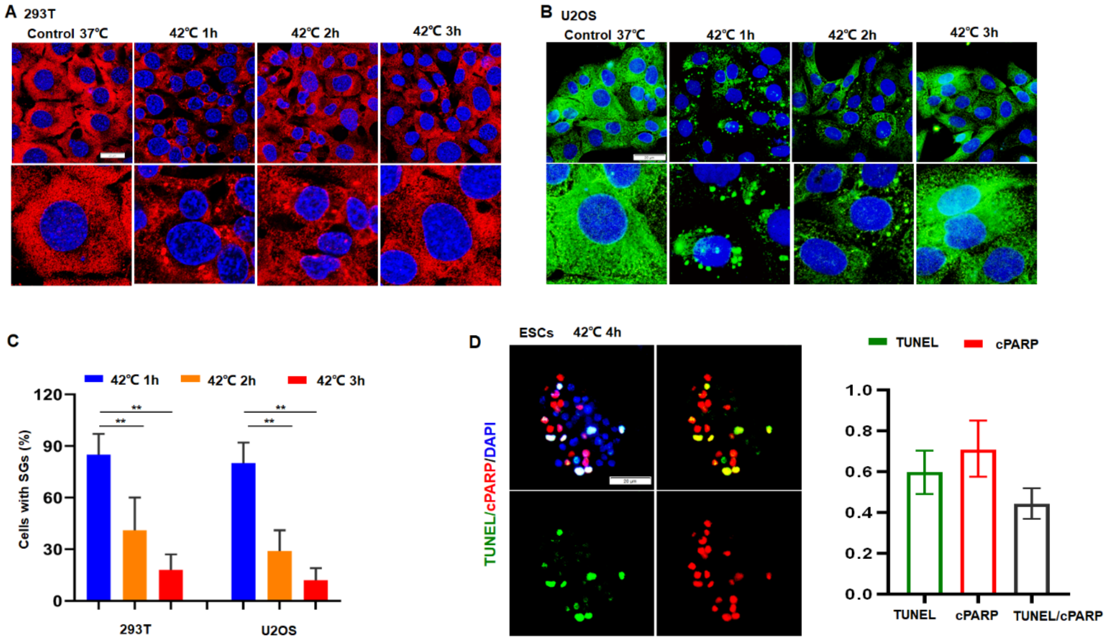

Figure S1. Dynamics of SGs in 293T and U2OS cells under prolonged stress.
(A) SGs in 293T cells under 42 ℃ stress for 1, 2 or 3 h, respectively. Scale bar, 20 μm.
(B) SGs in U2OS cells under 42 ℃ stress for 1, 2 or 3 h, respectively. Scale bar, 20 μm.
(C) Statistical analysis of G3BP1-SGs in 293T or U2OS cells under 42 ℃ stress for 1, 2 or 3 h. Mean ± SEM. $**p < 0.01$ (ANOVA test).
(D) TUNEL and cPARP staining in ESCs under 42 ℃ stress for 4 hours. Scale bar, 20 μm. Mean ± SEM.

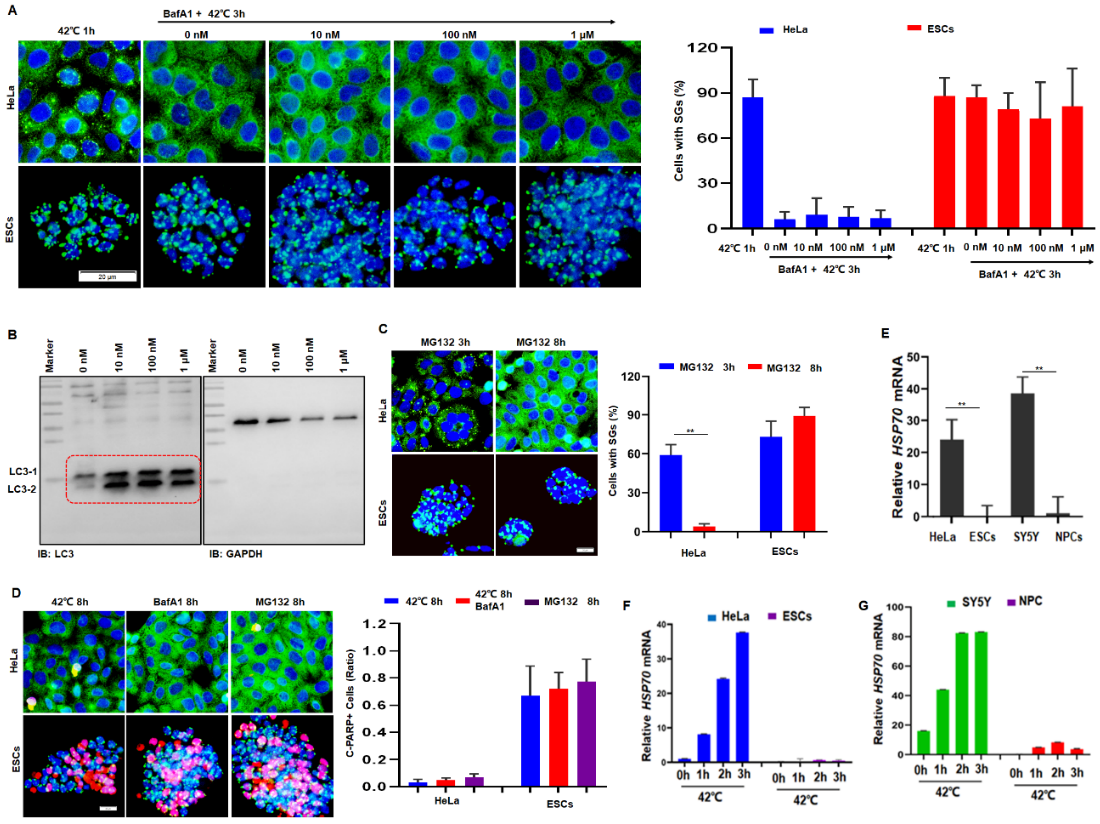

Figure S2. The majority of SGs disassemble in HeLa cells despite inhibition of autophagy or proteosome under prolonged stress.

(A) SGs in HeLa cells or ESCs treated with the indicated concentration of bafilomycin A1 exposed to 42 °C stress for 3h. Scale bar, 20 µm. Mean ± SEM.
(B) LC3 in HELA cells treated with the indicated concentration of bafilomycin A1 for 24h.
(C) SGs in HeLa cells or ESCs treated with 10 µM MG132 for 3 or 8h, respectively. Scale bar, 20 µm. Mean ± SEM. ***p* < 0.01 (Students *t*-test).
(D) cPARP staining in HeLa or ESCs treated with 1 µM bafilomycin A1 or 10 µM MG132 for 8h, or 42 °C stress for 8h. Scale bar, 20 µm. Mean ± SEM.
(E) Relative *HSP70* mRNA levels in HeLa, ESCs, SY5Y and NPCs. Mean ± SEM. ***p* < 0.01 (ANOVA test).
(F and G) Relative *HSP70* mRNA levels in HeLa, ESCs, SY5Y and NPCs after 42 °C stress for the indicated time. Mean ± SEM.

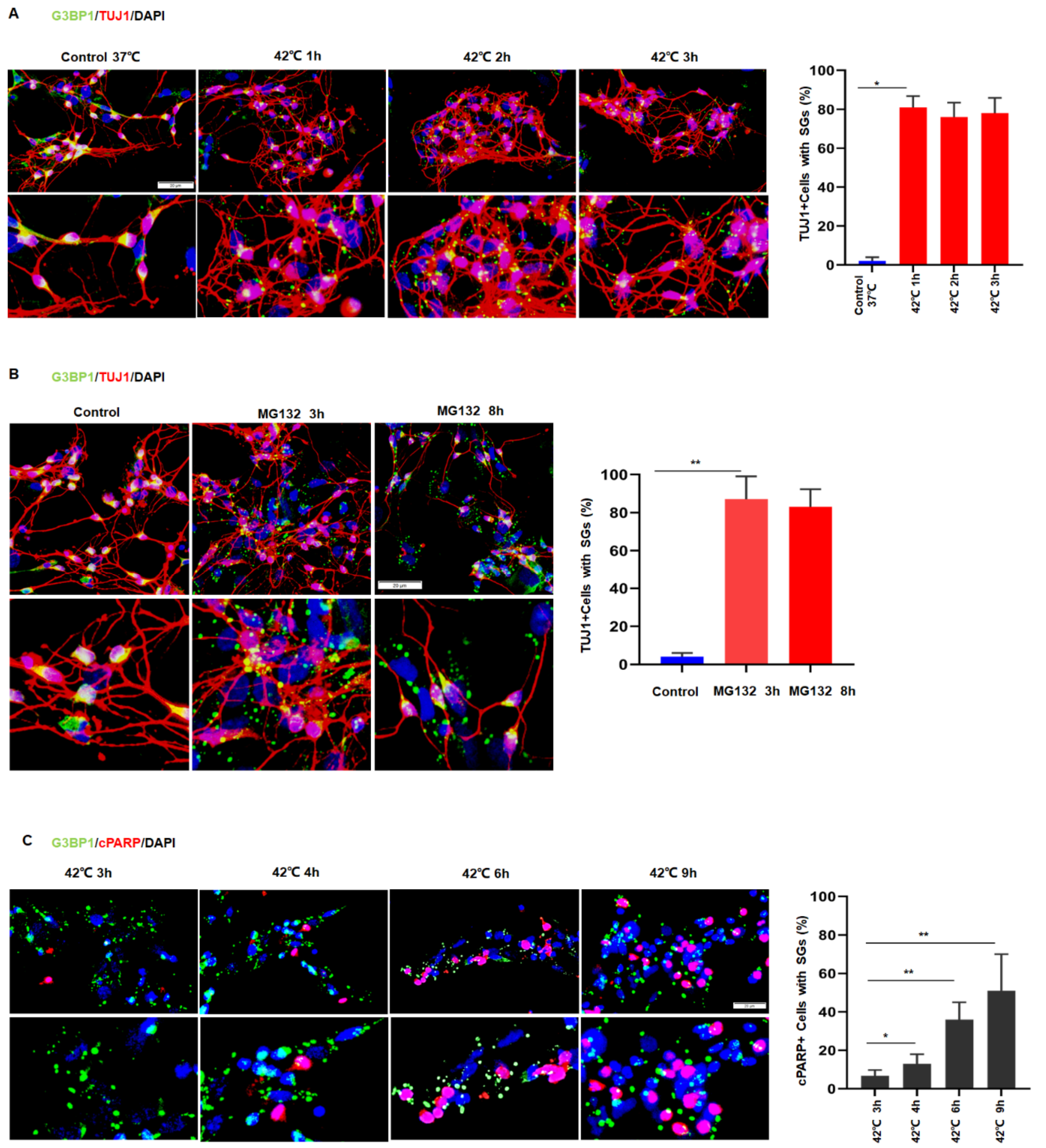

Figure S3. Persistent SGs led to cell apoptosis in primary cultured neurons under prolonged stress
(A) SGs in primary neurons under 42 °C stress for 1, 2 or 3 h, respectively. Scale bar, 20 µm. Mean ± SEM. ***p* < 0.01 (ANOVA test).
(B) SGs in primary neurons treated with 10 µM MG132 for 3 or 8h, respectively. Scale bar, 20 µm. Mean ± SEM. ***p* < 0.01 (ANOVA test).
(C) cPARP staining in primary neurons under prolonged stress. Scale bar, 20 µm. Mean ± SEM. **p* < 0.05, ***p* < 0.01 (ANOVA test).

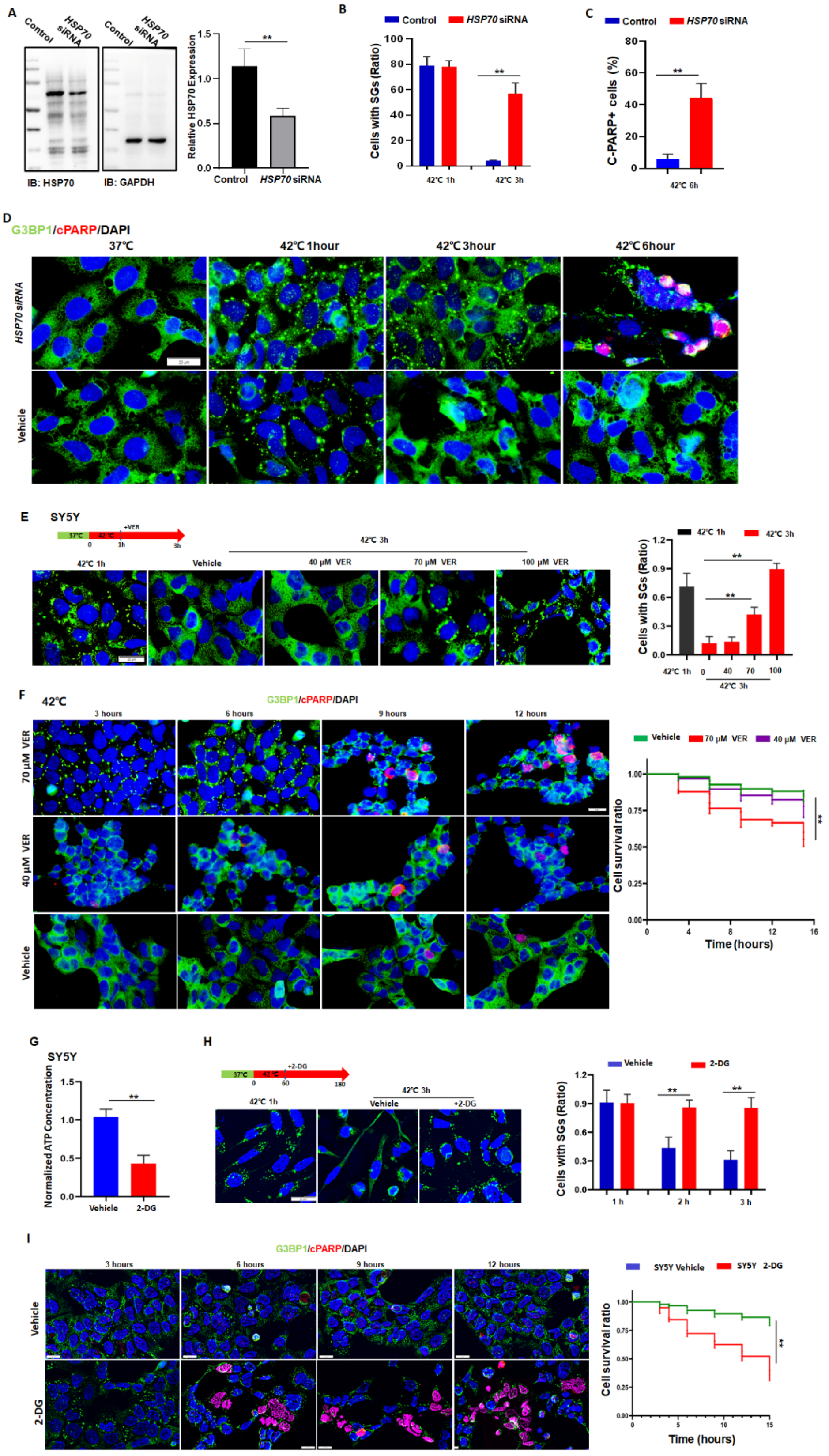
A
Control
HSP70 siRNA
IB: HSP70
IB: GAPDH
Relative HSP70 Expression
HSP70 siRNA
B
Control
HSP70 siRNA
Cells with SGs (Ratio)
42℃ 1h
42℃ 3h
C
C-PARP+ cells (%)
42℃ 6h
D
G3BP1/cPARP/DAPI
37℃
42℃ 1hour
42℃ 3hour
42℃ 6hour
HSP70 siRNA
Vehicle
E
SY5Y
+VER
42℃ 1h
Vehicle
42℃ 3h
40 μM VER
70 μM VER
100 μM VER
F
42℃
3 hours
6 hours
9 hours
12 hours
70 μM VER
40 μM VER
Cell survival ratio
Time (hours)
G
SY5Y
Normalized ATP Concentration
Vehicle
2-DG
H
+2-DG
1 h
2 h
3 h
I
SY5Y Vehicle
SY5Y 2-DG

Figure S4. HSP70 inhibition leads to Persistent SGs and cell apoptosis in neuroblastoma under prolonged stress

(A) HSP70 in SY5Y cells treated with control or *HSP70* siRNA. Mean ± SEM. $^{**}p < 0.01$ (Students *t*-test).

(B) SGs ratio in SY5Y cells treated with *HSP70* siRNA exposed to 42 ℃ stress for 1h, 3h. Mean ± SEM. $^{**}p < 0.01$ (ANOVA test).

(C and D) cPARP staining in SY5Y cells treated with *HSP70* siRNA exposed to 42 ℃ stress for the indicated time. Scale bar, 20 μm. Mean ± SEM. $^{**}p < 0.01$ (Students *t*-test).

(E) G3BP1 staining SGs in SY5Y cells exposed to 42 ℃ stress for 3 hours with or without addition of VER 2h. Scale bar, 20 μm. Mean ± SEM. $^{**}p < 0.01$ (ANOVA test).

(F) G3BP1 and cPARP staining and cell survival ratio in SY5Y cells exposed to 42 ℃ stress for the indicated time with or without addition of VER. Scale bar, 20 μm. Mean ± SEM. $^{**}p < 0.01$ (ANOVA test).

(G) ATP levels in SY5Y cells treated with or without 100 mM 2-DG 2h from three biological replicates. Mean ± SEM. ** $p < 0.01$ (Students *t*-test).

(H) SGs in SY5Y cells exposed to 42 ℃ stress for 1h, 2h, 3h with or without 2-DG. Scale bar, 20 μm. Mean ± SEM. ** $p < 0.01$ (Students *t*-test).

(I) G3BP1 and cPARP staining and cell survival ratio in SY5Y cells during 42 ℃ stress for the indicated time with or without 2-DG. Scale bar, 20 μm. Mean ± SEM. $^{**}p < 0.01$ (ANOVA test).

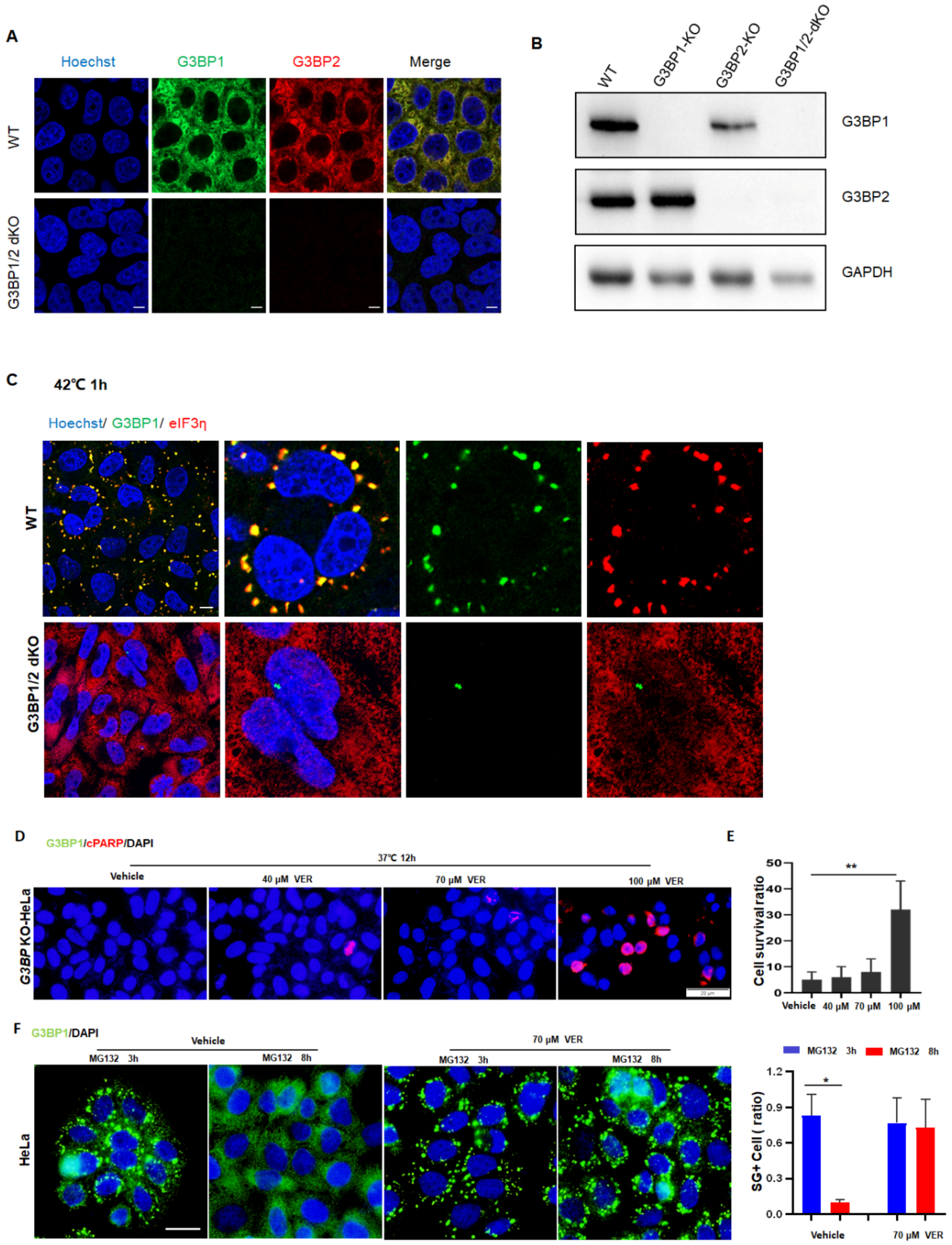

Figure S5. Establishment of G3BPs knock out HeLa cell lines
(A) G3BP1 and G3BP2 staining in HeLa cells and G3BP1/2 KO HeLa cells. Scale bar, 5 μm.
(B) Immunoblotting of G3BP1 and G3BP2 in G3BP1 or G3BP2 knock out HeLa cells. Original blots see Supplementary File S1. **(C)** G3BP1 and eIF3η staining in HeLa cells and G3BP1/2 KO HeLa cells after 42 °C stress for 1h. Scale bar, 10 μm. **(D and E)** cPARP staining in G3BP1/2 KO HeLa cells treated with or without addition of VER. Scale bar, 20 μm. Mean ± SEM. ***p* < 0.01 (ANOVA test). **(F)** SGs in HeLa cells treated with 10 μM MG132 and 70 μM VER for 3 or 8h, respectively. Scale bar, 15 μm. Mean ± SEM. **p* < 0.05 (ANOVA test).

File S1: Original Western Blot Data

Original Western Blot Data in Figure 2:

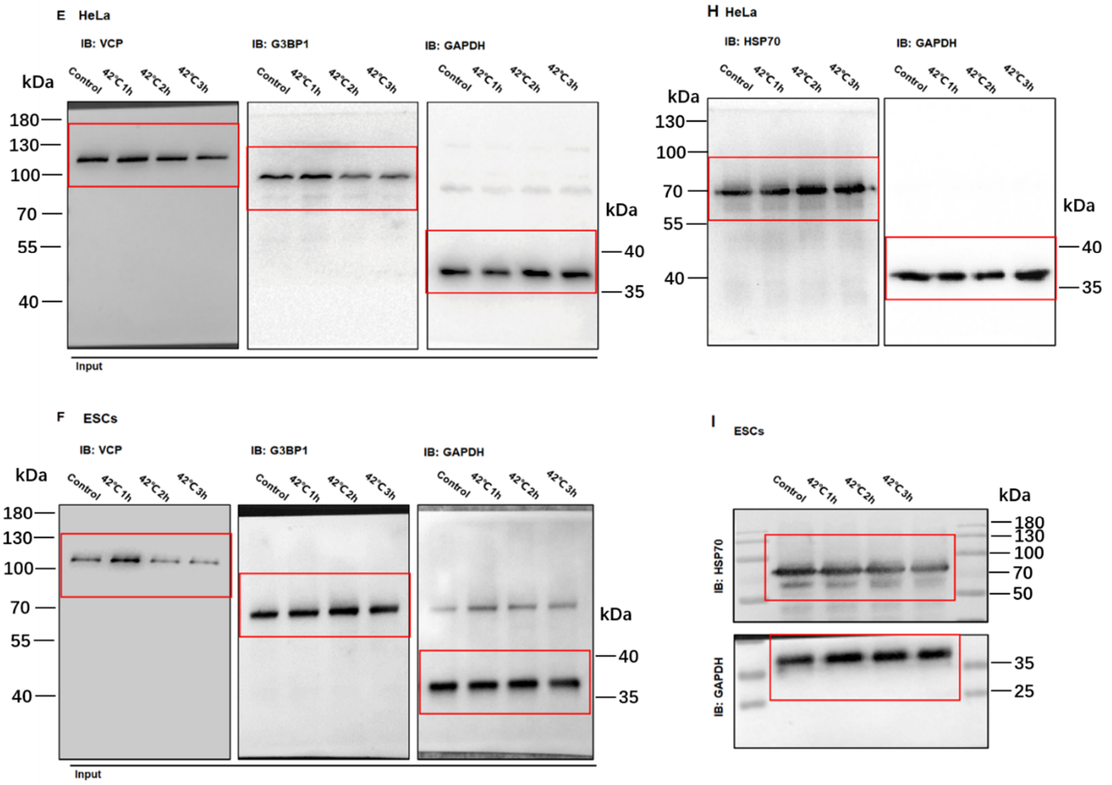

Original Western Blot Data in Figure 3:

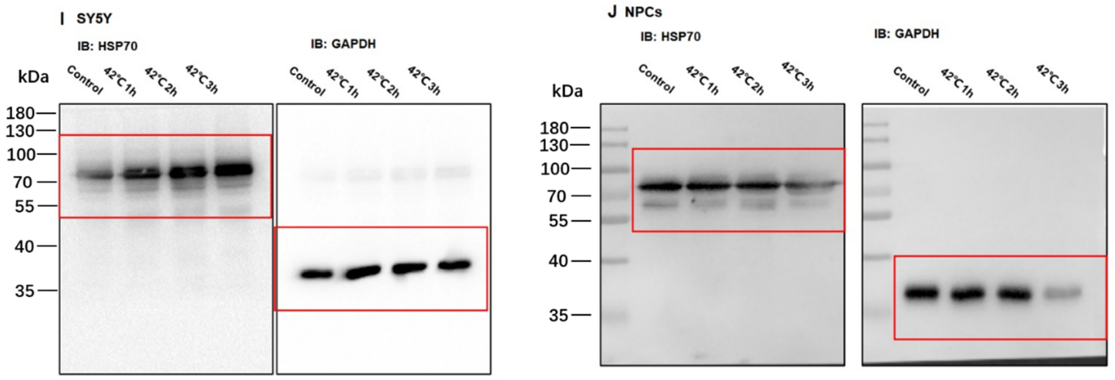

Corresponding to Figure 2 and Figure 3. Densitometry Readings/intensity Ratio:

**Densitometry reading/intensity Ratio**					
**HSP70/GAPDH**					
		**37℃**	**42℃1H**	**42℃2H**	**42℃3H**
**Figure 2H**	**HELA**	**1.082624**	**1.261499**	**1.597956**	**1.427598**
**Figure 2I**	**ESCs**	**0.956327**	**0.937959**	**0.880816**	**0.742041**
**Figure 3I**	**SY5Y**	**1.00157**	**1.22135**	**1.845369**	**2.343799**
**Figure 3J**	**NPC**	**1.024429**	**1.132559**	**1.244694**	**0.651982**

Original Western Blot Data in Figure S5:

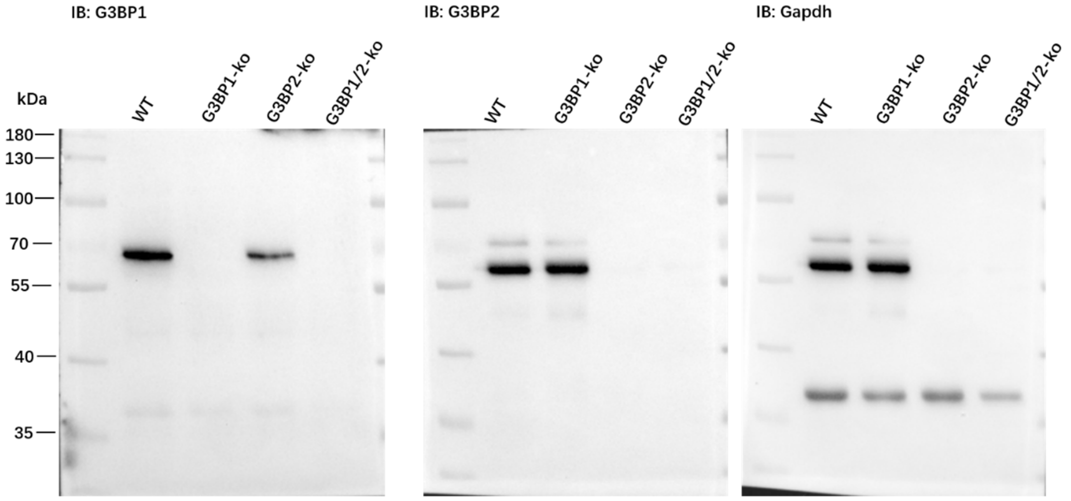

Table S1.
RESOURCES TABLE

REAGENT or RESOURCE	SOURCE	IDENTIFIER
Antibodies		
Anti-G3BP1	Abcam	Cat# ab181150<br>RRID: AB_2847886
Anti-G3BP1	Abcam	Cat# ab56574<br>RRID: AB_941699
Anti-CPARP	CST	Cat# 9548<br>RRID: AB_2160592
Anti-G3BP2	Novus Biologicals	Cat# NBP1-82977<br>RRID: AB_11016556
Anti-VCP	Abcam	Cat# ab109240<br>RRID: AB_10862588

Anti-DCP1A	Abnova	Cat# H00055802-M06<br>RRID: AB_530021
Anti-HSP70	Abcam	Cat# ab45133<br>RRID: AB_733035
Anti-Ubiquitin	Santa cruz	Cat# sc-8017<br>RRID: AB_628423
Anti-SOX2	Abcam	Cat# ab97959<br>RRID: AB_2341193
Anti-eIF3η	Santa cruz	Cat# sc-137214<br>RRID: AB_2277705
Anti-TUJ1	Santa cruz	Cat# sc-80005<br>RRID: AB_2210816
Anti-LC3	ABclonal	Cat# A19665<br>RRID: AB_2862723
Anti-GAPDH	Kangchen Biotech	Cat# KC-5G5<br>RRID: AB_2631280
Anti-mouse IgG, HRP-linked Antibody	CST	Cat# 7076S<br>RRID: AB_330924
Anti-rabbit IgG, HRP-linked Antibody	CST	Cat# 7074S<br>RRID: AB_2099233
Donkey anti-Mouse IgG(H+L) highly Cross-Adsorbed secondary Antibody, Alexa Fluor 594	Thermo Fisher Scientific	Cat# A-21203<br>RRID: AB_141633
Donkey anti-Mouse IgG(H+L) highly Cross-Adsorbed secondary Antibody, Alexa Fluor 488	Thermo Fisher Scientific	Cat# A-21202<br>RRID: AB_141607
Donkey anti-Rabbit IgG(H+L) highly Cross-Adsorbed secondary Antibody, Alexa Fluor 594	Thermo Fisher Scientific	Cat# A-21207<br>RRID: AB_141637
Donkey anti-Rabbit IgG(H+L) highly Cross-Adsorbed secondary Antibody, Alexa Fluor 488	Thermo Fisher Scientific	Cat# A-21206<br>RRID: AB_2535792
ANTI-FLAG M2 Magnetic Beads	sigma	Cat# M8823<br>RRID: AB_2637089
Chemicals		
2-DG	selleck	Cat# S4701
VER155008	selleck	Cat# S7751
Bafilomycin A1	MCE	Cat# HY-100558
MG132	MCE	Cat# HY-13259
Oligonucleotides		
Primer for constructing pCMV7.1-G3bp1-GFP plasmid forward: 5'-GACGATGACAAGCTTATGGTTATGGAGAAGCCTAGT-3'	This paper	N/A
Primer for constructing pCMV7.1-G3bp1-GFP plasmid reverse 5'-CTCCTCGCCCTTGCTCACCAT -3'	This paper	N/A
Primer for G3bp1-KO gRNA forward: 5'-CACCGaaattcccgcccgaccagca -3'	This paper	N/A
Primer for G3bp1-KO gRNA reverse: 5'-AAACtgctggtcgggcgggaatttC -3'	This paper	N/A
Primer for G3bp1-KO gRNA forward: 5'-CACCGtagtcccctgctggtcgggc -3'	This paper	N/A
Primer for G3bp1-KO gRNA reverse: 5'-AAACgcccgaccagcaggggactaC -3'	This paper	N/A
Primer for G3bp2-KO gRNA forward: 5'-CACCGccgccctacaagcagcggac -3'	This paper	N/A
Primer for G3bp2-KO gRNA reverse: 5'-AAACgtccgctgcttgtagggcggC -3'	This paper	N/A

Primer for G3bp2-KO gRNA forward: 5'-CACCGcgccctacaagcagcggact -3'	This paper	N/A
Primer for G3bp2-KO gRNA reverse: 5'-AAACagtccgctgcttgtagggcgC -3'	This paper	N/A
Primer for *HSP70* in vitro transcription forward: 5'-GCGAGGCGGACAAGAAGAA -3'	This paper	N/A
Primer for *HSP70* in vitro transcription reverse: 5'-GATGGGGTTACACACCTGCT -3'	This paper	N/A
Primer for *GAPDH* in vitro transcription forward: 5'-ACCACAGTCCATGCCATCAC -3'	This paper	N/A
Primer for *GAPDH* in vitro transcription reverse: 5'-TCCACCACCCTGTTGCTGTA -3'	This paper	N/A
